# Modulation of Sodium and Ammonia Transporters in the Context of Viral Gill Diseases in Common Carp (*Cyprinus carpio*)

**DOI:** 10.1111/jfd.14133

**Published:** 2025-04-23

**Authors:** Maria Zawisza, Muhammad Abdullah, Magdalena Marcinkowska, Marek Matras, Krzysztof Rakus, Mikolaj Adamek

**Affiliations:** ^1^ Department of Evolutionary Immunology Institute of Zoology and Biomedical Sciences, Faculty of Biology, Jagiellonian University Krakow Poland; ^2^ Doctoral School of Exact and Natural Sciences, Jagiellonian University Krakow Poland; ^3^ Fish Disease Research Unit, Institute for Parasitology, University of Veterinary Medicine Hannover Hannover Germany; ^4^ Department of Parasitology and Invasive Diseases Bee Diseases and Aquatic Animal Diseases, National Veterinary Research Institute Pulawy Poland

**Keywords:** ammonia transporters, carp edema virus, common carp, cyprinid herpesvirus‐3, Na^+^/K^+^‐ATPases, osmoregulation

## Abstract

Osmoregulation and ammonia removal are among key physiological processes that take place in gills and affect fish homeostasis and well‐being. These processes can be disrupted by numerous environmental factors, but also by viral infections, especially those leading to severe gill disorders. The mechanisms of how viruses disrupt osmoregulation and ammonia removal in fish have not been extensively studied. We propose further exploration of the molecular and functional basis of viral induced gill disorders by studying the gene expression and enzyme activity of Na^+^/K^+^‐ATPases (NKA) and ammonia transporters in the gills and kidney of common carp during infection with two viruses: carp edema virus (CEV) and cyprinid herpesvirus 3 (CyHV‐3). In the case of NKA, the expression of subunit α of selected NKA was affected by both viruses; however, no discernible trends were observed in the gills and kidneys. The enzyme activity of NKA was significantly reduced in the gills during infection with both CEV and CyHV‐3. Moreover, our immunohistochemical studies showed that during infection with CEV and CyHV‐3, NKA‐rich cells are transferred from the primary lamellae to the more superficial space and to the secondary lamellae of the gills. In the case of ammonia transporters, both CEV and CyHV‐3 infection resulted in downregulation of the expression of major transporters *gdh1*, *Rhag*, and *Rhbg*, allowing us to partly explain the ammonia accumulation in blood observed during infection with these viruses. This study highlighted that virus induced gill disorders may lead to disruption of osmoregulation and ammonia removal by dysregulating the expression and activity of NKA and ammonia transporters.

## 1 Introduction

Fish gills are multifunctional organs responsible for a variety of physiological processes, including gas exchange, osmoregulation, ion exchange, acid–base balance, ammonia excretion, modification of circulating metabolites, and immune responses (Evans et al. 2005; Rességuier et al. 2020; Rombough 2007; Zawisza, Chadzinska et al. 2024). Osmoregulation and ion exchange are suggested to be the primary functions of gills during fish development, as ion exchange shifts to gills earlier than gas exchange in the larvae of zebrafish ( *Danio rerio* ) (Rombough 2007) and rainbow trout ( *Oncorhynchus mykiss* ) (Fu et al. 2010). Ion exchange differs between freshwater and marine fish species due to different salt and osmotic gradients in the environment. Marine fish species absorb water to counteract osmotic loss and actively secrete ions Na^+^ and Cl^−^ across the gill epithelium to maintain ionic balance. In freshwater fish species, this process acts in the opposite direction. Freshwater fish live in a hypoosmotic environment, so they are subjected to a constant gain of water by osmosis and actively transport Na^+^ and Cl^−^ from the environment by various ion transporters. They also produce diluted urine to counter the water influx (Evans et al. 2005; Marshall 2002). Na^+^/K^+^‐ATPases (NKA) in the gill epithelium play the most crucial role in ion regulation in both marine and freshwater fish species (Evans et al. 2005; Marshall 2002). Na^+^/K^+^‐ATPases are heterodimers and consist of a catalytic α subunit and a smaller glycosylated β subunit, encoded by separate genes (Blanco and Mercer 1998; Morth et al. 2011). In the gills of freshwater fish species, these membrane‐bound enzymes are located in the basolateral membrane of the ionocytes (mitochondria‐rich cells [MRCs], also called chloride cells [CCs]) and actively transport Na^+^ from the cells into the blood and K^+^ from the blood into the cells, allowing sodium uptake by fish. The initial steps of sodium transport are facilitated by V‐type H^+^ ATPases at the apical membrane, which generate a large transmembrane potential necessary for the passive uptake of Na^+^ ions. Na^+^ ions can also enter the cells through epithelial sodium channels (ENaC) using the electrochemical gradient. Additionally, there is evidence supporting the involvement of Na^+^/H^+^ exchangers (NHE), located in the apical membrane of the cells, in the uptake of Na^+^ ions. Tight junctions help minimise paracellular ion loss under homeostatic conditions, ensuring efficient regulation of ion balance (Marshall 2002).

Beyond osmoregulation another crucial gill function is nitrogenous waste excretion, as gills are the primary site of ammonia excretion in fish. Even though, fish are more tolerant to ammonia when compared to mammals, ammonia is still toxic and can be harmful especially for the central nervous system (Randall and Tsui 2002). A few mechanisms are involved in ammonia excretion, which include passive diffusion of NH_3_ and/or NH_4_^+^, and the transport of ammonia through Na^+^/H^+^ exchangers (Evans et al. 2005). In this process NH_4_^+^ is substituted for H^+^ when needed (Wright and Wood 2009, 2012). Another group of proteins playing a critical role in ammonia transport in fish are Rhesus (Rh) glycoproteins. These integral membrane proteins belong to the ammonia transporter family and facilitate the movement of ammonia across cell membranes, enabling fish to regulate nitrogen excretion and maintain acid–base balance (Wright and Wood 2009). Rh glycoproteins are particularly vital in gill tissues, where they help excrete ammonia directly into the water, either as NH_3_ or NH_4_^+^, depending on the environmental pH. Their efficient function was shown to be crucial for the survival of fish, especially in ammonia‐rich or variable pH habitats (Wright and Wood 2009, 2012).

NKA and ammonia transporters in gills are expressed in specialised epithelial cells and their activity can be modulated by internal and external factors, including water parameters such as temperature, salinity, pH, hardness and ammonia concentration (Einarson 1993; Handeland et al. 1998; Lemaire et al. 2002; Vargas‐Chacoff et al. 2020, 2018). Moreover, NKA and ammonia transporters can also be actively modulated during infections (Baldissera et al. 2019; Deane and Woo 2005; Fjelldal et al. 2020; Meyer et al. 2002). There are two viral diseases which have the biggest impact on common carp ( *Cyprinus carpio* ) aquaculture worldwide: koi sleepy disease (KSD) induced by carp edema virus (CEV) (Jung‐Schroers et al. 2015; Adamek et al. 2017; Machat et al. 2021; Adamek et al. 2021; Zawisza, Chadzinska et al. 2024) and koi herpesvirus disease (KHVD) induced by Cyvirus cyprinidallo3 also known as cyprinid herpesvirus 3 (CyHV‐3) (Waltzek et al. 2005; Negenborn et al. 2015; Rakus et al. 2013; Boutier et al. 2015, 2019). Infections by both CEV and CyHV‐3 result in changes in the blood parameters (ammonia accumulation, osmotic balance disruption) which indicate impairment of gill function of infected fish (Adamek et al. 2021; Negenborn et al. 2015; Rakus et al. 2013; Zawisza, Rebl et al. 2024). CEV induces hypoxia, hyponatremia and hyperammonemia combined with swelling and necrosis of gill tissue and leads to mortality of up to 100% of infected fish (Adamek et al. 2021; Pikula et al. 2021; Zawisza, Rebl et al. 2024). However, the mechanisms responsible for this have not been fully elucidated yet. Importantly, high mortality of fish due to CEV infection can be prevented by salt treatment which allows fish to restore sodium concentration and balance ammonia levels, clearly indicating that gill dysfunction is responsible for high mortality during KSD (Adamek et al. 2021; Miyazaki et al. 2005; Zawisza, Rebl et al. 2024). In the case of CyHV‐3, a systemic infection is observed, which results in strong histopathological changes in gills, but also other organs (Miyazaki et al. 2008; Pikarsky et al. 2004; Rakus et al. 2013). In gills, CyHV‐3 infection induces detachment and degeneration of epithelial cells which can impair ion uptake (Negenborn et al. 2015). It has been suggested that tissue damage in gills and kidney during CyHV‐3 infection leads to disruption of osmoregulation. For example, renal tubule damage in the kidney has been suggested as a cause of increased osmolarity of urine and sodium escape (Negenborn et al. 2015). As a consequence, sodium concentration in blood is significantly decreased, which could be one of the major causes of fish mortality during CyHV‐3 infection (Miwa et al. 2015; Negenborn et al. 2015). As previous studies did not fully explain the mechanisms of osmoregulation disruption, we propose further exploration of the molecular and functional basis of gill disorders in common carp caused by CEV and CyHV‐3. Our study focused on the α subunits of NKA and ammonia transporters which have not been extensively studied during viral infections in fish so far.

## 2 Materials and Methods

### 2.1 Fish

Naïve common carp of Prerov scaly strains (PS) and koi were obtained as feeding yolk sac fry from the University of South Bohemia in Ceske Budejovice, Faculty of Fisheries and Protection of Waters, located in Vodnany, Czech Republic. Fish were raised and kept under virus‐ and parasite‐free conditions in an indoor aquaculture recirculation system at 20°C. Fish were fed a commercial feed (Perla Plus, Skretting Norway) at a ratio of 1% body weight per day.

### 2.2 Sequence Alignment and Phylogenetic Analysis

The orthologs of α subunits of different NKAs and ammonia transporters were identified by data‐mining from the National Center for Biotechnology Information (NCBI, https://www.ncbi.nlm.nih.gov/). Amino acid sequences of α subunits of NKAs and ammonia transporters from common carp, zebrafish and Nile tilapia ( *Oreochromis niloticus* ) were aligned, and phylogenetic trees were constructed based on the full‐length amino acid sequences using the maximum likelihood method with approximate likelihood‐ratio test (aLRT) for branches using Phylogeny.fr Robust Phylogenetic Analysis For The Non‐Specialist (Dereeper et al. 2008).

### 2.3 Ethics

All experiments were carried out in accordance with national and international regulations for experimentation with animals under the approval of the Local Ethical Committee in Lublin, Poland (no. 32/2020) and the Lower Saxony State Office for Consumer Protection and Food Safety (LAVES), Oldenburg, Germany (no. 33.19‐425 2‐04‐16/2144 and 33.8‐425 02‐04‐20/3414).

### 2.4 Infection Experiment

Experimental infection with carp edema virus was performed by exposure of naïve koi carp (*n* = 8; mean weight 93.7 ± 27.1 g) to clinically affected, virus‐shedding donor fish (koi fingerling *n* = 3) obtained for research and diagnostic purposes from an outbreak of the disease. For this study, koi infected with CEV from genogroup IIa were used. For the mock‐infected group, naïve SPF koi carp (*n* = 8) were cohabitated with non‐infected SPF koi from a different cohort. At 6 days post infection (dpi) fish from CEV‐infected and mock‐infected groups were euthanised by immersion in 0.5 g/L MS‐222 (Sigma, USA) and subsequent decapitation.

For CyHV‐3 studies, samples were obtained from a previously performed experiment (Adamek et al. 2022) according to 3R rules. In short, experimental infection with CyHV‐3 was performed by the bath challenge with a hyper‐virulent wild type of Polish isolate of CyHV‐3 (Adamek et al. 2022). Naïve common carp from the Prerov scaly strain (mean weight 125.6 ± 54.6 g) were exposed to CyHV‐3 at a final concentration of 1.4 × 10^3^ PFU/mL in small tanks with 40 L water at 23°C ± 1°C for 2 h with constant aeration (Adamek et al. 2022). Mock‐infected control Prerov scaly carp were exposed to culture medium without virus (Adamek et al. 2022). At 7 dpi, fish from CyHV‐3‐infected (*n* = 6) and mock‐infected (*n* = 6) groups were euthanised by immersion in 0.5 g L^−l^ MS‐222 (Sigma, USA).

### 2.5 Sample Collection

Blood was collected with S‐Monovette (Sarstedt, Germany) from the dorsal aorta. Half of the second gill arch and a fragment of the trunk kidney were sampled and directly transferred to RNAlater and stored at −80°C for further analysis. The third gill arch was used for the ATPase activity assay; for this, it was homogenised in SEID buffer (250 mmol/L sucrose, 10 mmol/L EDTANa_2_, 50 mmol/L imidazole and 0.1% sodium deoxycholate) and centrifuged at 5000× *g* for 30 s, and the supernatant was immediately frozen in liquid nitrogen. The second half of the second gill arch was used for immunohistochemistry and was collected and fixed with 4% phosphate buffered formaldehyde solution.

### 2.6 Blood Parameter Analyses

100 μL of blood was used for blood parameter analyses with the OPTI CCA‐TS blood gas analyser (OPTI Medical Systems, USA) as described previously (Adamek et al. 2021). OPTI sensor cassettes E‐Ca were used to detect sodium (Na^+^) and potassium (K^+^) concentrations (Adamek et al. 2021). The rest of the blood was centrifuged for 15 min at 600× *g* at 4°C; plasma was collected and stored at −80°C. Sodium levels below 100 mmol/L were determined with a flame photometer (Bayer Diagnostics, Germany). Ammonia levels were detected by a photometric test (LT Sys, Germany) within 1 week after plasma collection (Adamek et al. 2021).

### 2.7 DNA Isolation

DNA was isolated from gills and kidney tissues. Gills and/or kidney were homogenised in a Tissuelyser II (Qiagen, Germany) and then DNA was extracted using the QIAamp DNA Mini Kit (Qiagen, Germany) according to the manufacturer's protocol.

### 2.8 RNA Isolation and cDNA Synthesis

RNA was isolated from gills and kidney as described previously (Adamek et al. 2021) using TRI reagent (Sigma, USA) according to the manufacturer's protocol. In order to purify samples from any genomic DNA contamination, samples were subjected to additional DNase I (2 U) digestion (Thermo Fisher Scientific, USA). cDNA was synthesised from 300 ng of RNA using the Maxima First Strand cDNA Synthesis Kit (Thermo Fisher Scientific, USA).

### 2.9 Real‐Time Quantitative PCR

#### 2.9.1 Viral Load Analysis

In order to determine viral load in gills and kidney, a probe‐based RT‐qPCR (double labelled probe‐based) was performed. In the case of CEV, the p4a gene was used as a target to quantify viral load (Matras et al. 2017) and in the case of CyHV‐3, the ORF89 gene was studied (Gilad et al. 2004).

#### 2.9.2 Gene Expression Analysis

For gene expression study a Maxima SYBR Green RT‐qPCR was performed as described previously (Adamek et al. 2022). 40S ribosomal protein S11 and elongation factor 1 alpha were used as reference genes. Gene expression is presented as number of copies of the target gene normalised against 1 × 10^5^ copies of the reference genes (Adamek et al. 2022). Primers are presented in Table S1.

### 2.10 Immunofluorescent Staining

Gills fixed in 4% phosphate‐buffered formaldehyde solution were dehydrated and embedded in paraffin and cut into 4 μm thick sections. Slides were deparaffinised, rehydrated and an antigen retrieval protocol was performed with sodium citrate buffer (10 mM of sodium citrate, 0.05% Tween 20, pH = 6). Slides were blocked with 1% BSA and 0.1% Triton X. Next, slides were incubated with rabbit polyclonal primary antibody against NKA (#3010, Cell Signalling Technology, USA) at 1:300 in Pierce Immunostain Enhancer (Thermo Fisher Scientific, USA). After overnight incubation at 4°C, slides were washed with PBS and incubated with secondary antibody CyTM goat anti‐rabbit (Thermo Fisher Scientific, USA) at 1:500 in Pierce Immunostain Enhancer. In addition, two types of controls were used: (i) the primary antibody was replaced by the blocking solution, (ii) during the primary antibody testing and selection process, an isotype control was performed by replacing the primary antibody with rabbit control IgG (#02–6102 Thermo Fisher Scientific, USA). Slides were mounted with RotiMount with DAPI (Roth, Germany). Images were captured on Keyence BZ‐X810 in the case of CEV or on ZEISS AXIO Imager M2 in the case of CyHV‐3 and analysed using ImageJ.

### 2.11 NKA Activity Analysis

NKA activity was measured according to the method by McCormick (McCormick 1993). The ouabain‐sensitive hydrolysis of adenosine triphosphate (ATP) is enzymatically coupled to the oxidation of nicotinamide adenine dinucleotide (reduced from NADH), which is measured directly in a microplate reader. Two assay mixtures (Solution A, Solution B) were prepared for activity measurement. Solution A, consisting of 4 U lactate dehydrogenase (LDH) mL^−1^, 5 U pyruvate kinase (PK) mL^−1^, 2.8 mM phosphoenolpyruvate (PEP), 0.7 mM ATP, 0.22 mM NADH and 50 mM imidazole, was prepared before the assay and stored at 4°C (biochemical reagents from Sigma Aldrich, Roche, Roth. Germany). Solution B is the same as above but also contains 0.5 mM ouabain. A salt solution containing 189 mM NaCl, 10.5 mM MgCl, 42 mM KCl and 50 mM imidazole was prepared in advance. Assay solutions (A and B) and salt solutions were then mixed separately in a 3:1 ratio and kept on ice. Assay solutions were warmed to room temperature prior to use.

Samples were taken out of −80°C immediately prior to the experiment and were thawed and kept on ice throughout the procedure. A 96‐well microtiter plate was used for analysis. 10 μL of each sample was added to the microtiter plate in duplicate for both with ouabain and without ouabain (4 wells per sample, 2 with ouabain, 2 without ouabain). Finally, 200 μL of assay solutions A and B were added to the respective wells. The microtiter plate was then placed in the plate reader, and the rate of NADH disappearance was measured at 340 nm absorbance for 10 min in the BMG FLUOstar Optima Microplate Reader. The linear rate from 5 to 10 min in each pair of duplicate wells was determined, and NKA activity was calculated as the difference in ATP hydrolysis in the absence and presence of ouabain, the results are expressed in U/mL and reflect the amount of ADP generated per milligram of protein (McCormick 1993).

### 2.12 Statistical Analysis

Statistical analysis was done in SigmaPlot 12.5 software (Systat Software). Normal distribution of the samples was assessed by the Shapiro–Wilk test. Significant differences (*p* ≤ 0.05) in blood parameters and gene expression studies were assessed using Student's *t* test in cases when the data were normally distributed or by the Mann–Whitney test when the data were not normally distributed. The data are presented as *n* = 8 individual values for CEV infection and *n* = 5 or *n *= 6 individual values for CyHV‐3 infection on a box and whiskers plot.

## 3 Results

### 3.1 Phylogenetic Analysis of Common Carp, Zebrafish and Nile Tilapia NKA and Ammonia Transporters

#### 3.1.1 NKA Phylogenetic Tree for Amino Acid Sequence

**FIGURE 1:**
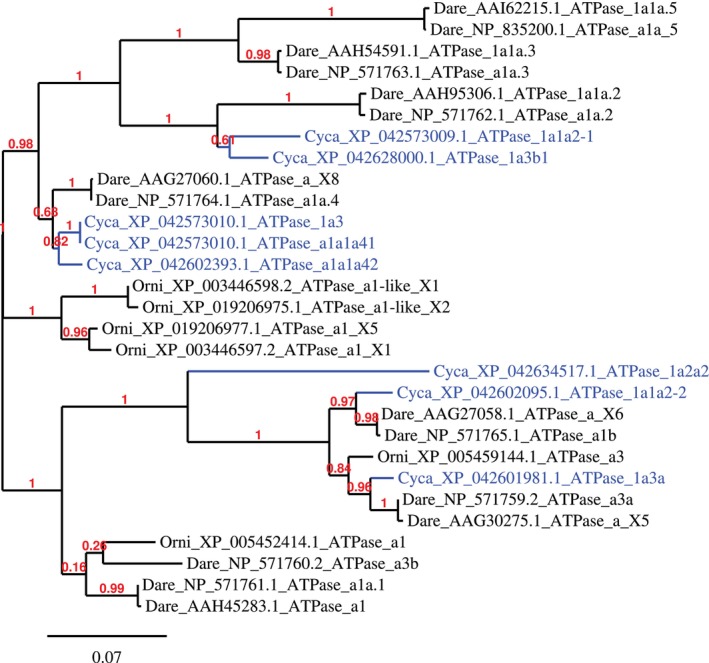
Phylogenetic tree based on amino acid sequence of α subunits of Na^+^/K^+^‐ATPases from common carp, zebrafish and Nile tilapia. Marked in blue are sequences studied in this paper. Numbers in red are aLRT branch support values. Branch length is proportional to the number of substitutions per site. Phylogenetic tree using maximum likelihood method was created by Phylogeny.fr.

Phylogenetic analysis using the maximum likelihood method revealed 5 clusters of NKA α subunits (Figure 1). Most of the NKA from Nile tilapia, with the exception of ATPase a3 and a1, clustered together. Common carp NKA 1a1a2–1 and 1a3b1 clustered with zebrafish NKA 1a1a.5, a1a_5, 1a1a.3, a1a.3, 1a1a.2, and a1a_2. Another cluster consists of zebrafish NKA a_X8, a1a.4, and common carp NKA 1a3, a1a1a41, and a1a1a42. Common carp NKA 1a2a2, 1a1a2–2, 1a3a, alongside zebrafish a_X6, a1b, a3a, a_X5 clustered with Nile tilapia a3. Last but not least, Nile tilapia NKA a1 clustered with zebrafish a3b, a1a.1, and a1 (Figure 1).

#### 3.1.2 Ammonia Transporters Phylogenetic Tree for Amino Acid Sequence

**FIGURE 2:**
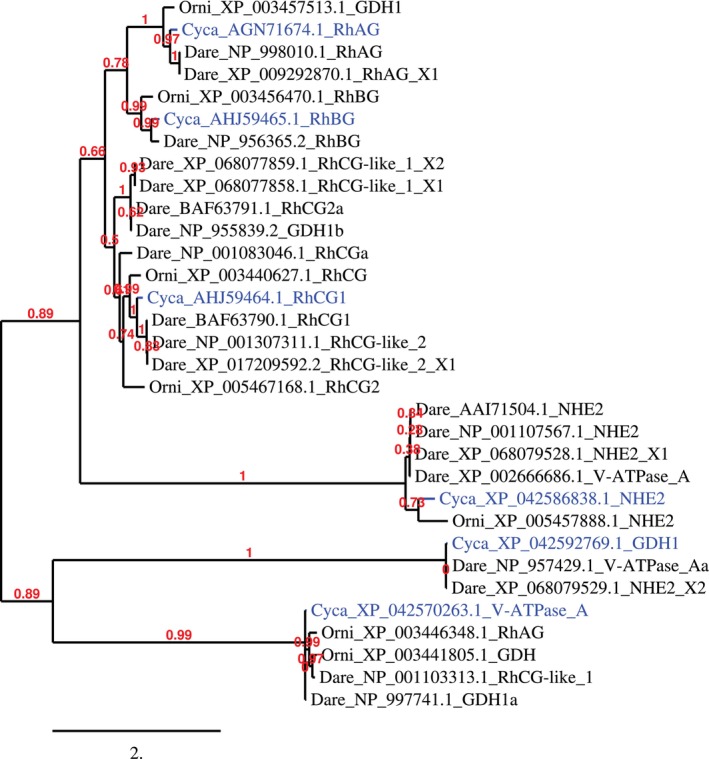
Phylogenetic tree based on amino acid sequence of ammonia transporters from common carp, zebrafish and Nile tilapia. Marked in blue are sequences studied in this paper. Numbers in red are aLRT branch support values. Branch length is proportional to the number of substitutions per site. Phylogenetic tree using maximum likelihood method was created by Phylogeny.fr.

Phylogenetic analysis using the maximum likelihood method revealed 4 clusters (Figure 2). The first cluster consists mostly of Rh factors and includes Nile tilapia GDH1, RhBG, RhCG, RhCG2, zebrafish RhAG, RhAG isoform X1, RhBG, RhCG‐like_1 X1 and X2, RhCG2a, GDH1b, RhCGa, RhCG1, RhCG‐like_2 and RhCG‐like_2 X1, as well as common carp transporters RhAG, RhBG, RhCG1. The second cluster includes transporters from zebrafish: NHE2, NHE2 X1, V‐ATPase_A, Nile tilapia and common carp NHE2. Common carp GDH1 and zebrafish V‐ATPase_Aa and NHE2 X2 form the third cluster of ammonia transporters. The last cluster is made of common carp V‐ATPase_A, Nile tilapia RhAG and GDH and zebrafish RhCG‐like_1 and GDH1a (Figure 2).

### 3.2 Active Domains of NKA and Ammonia Transporters

Active domains and the chromosome on which particular genes are located are all present in a Table 1. In analysed genes encoding α subunits of NKAs, the active domain was ATPase‐IIC_X‐K sodium or proton efflux–potassium uptake antiporter (present in ATPase NKA 1a1a2–1, NKA 1a1a2–2, NKA 1a2a2, NKA 1a3, NKA 1a3a, NKA 1a3b1, NKA a1a1a41, NKA a1a1a42). In ammonia transporters, domains such as V‐type (H^+^)‐ATPase V1, A subunit were present in VA 6v1, glutamate dehydrogenase/leucine dehydrogenase in GDH1, ammonium transporter superfamily in RhAG, RhACG1, RhBG and Na^+^/H^+^ exchanger in NHE2 (Table 1).

**TABLE 1:** Active domains of proteins encoded by studied genes in common carp and chromosome on which genes are located identified by data mining from the National Center for Biotechnology Information (NCBI, https://www.ncbi.nlm.nih.gov/).

Gene	Protein	Chromosome	Active domain of protein
Sodium transporters			
*nka 1a1a2–1*	NKA 1a1a2–1	B1	ATPase‐IIC_X‐K sodium or proton efflux
*nka 1a1a2–2*	NKA 1a1a2–2	A1	ATPase‐IIC_X‐K sodium or proton efflux
*nka 1a2a2*	NKA 1a2a2	A2	ATPase‐IIC_X‐K sodium or proton efflux
*nka 1a3*	NKA 1a3	B1	ATPase‐IIC_X‐K sodium or proton efflux
*nka 1a3a*	NKA 1a3a	B10	ATPase‐IIC_X‐K sodium or proton efflux
*nka 1a3b1*	NKA 1a3b1	A16	ATPase‐IIC_X‐K sodium or proton efflux
*nka a1a1a41*	NKA a1a1a41	B1	ATPase‐IIC_X‐K sodium or proton efflux
*nka a1a1a42*	NKA a1a1a42	A1	ATPase‐IIC_X‐K sodium or proton efflux
Ammonia transporters			
*va6v1*	VA 6v1	A24	V‐type (H+)‐ATPase V1, A subunit
*gdh1*	GDH1	B13	Glutamate dehydrogenase/leucine dehydrogenase
*Rhag*	RhAG	B20	Ammonium transporter superfamily
*Rhcg1*	RhCG1	A25	Ammonium transporter superfamily
*Rhbg*	RhBG	A16	Ammonium transporter superfamily
*nhe2*	NHE2	B9	Sodium/hydrogen exchanger family

### 3.3 Viral Load and Na^+^, K^+^ and Ammonia Concentration Upon CEV and CyHV‐3 Infection

**FIGURE 3:**
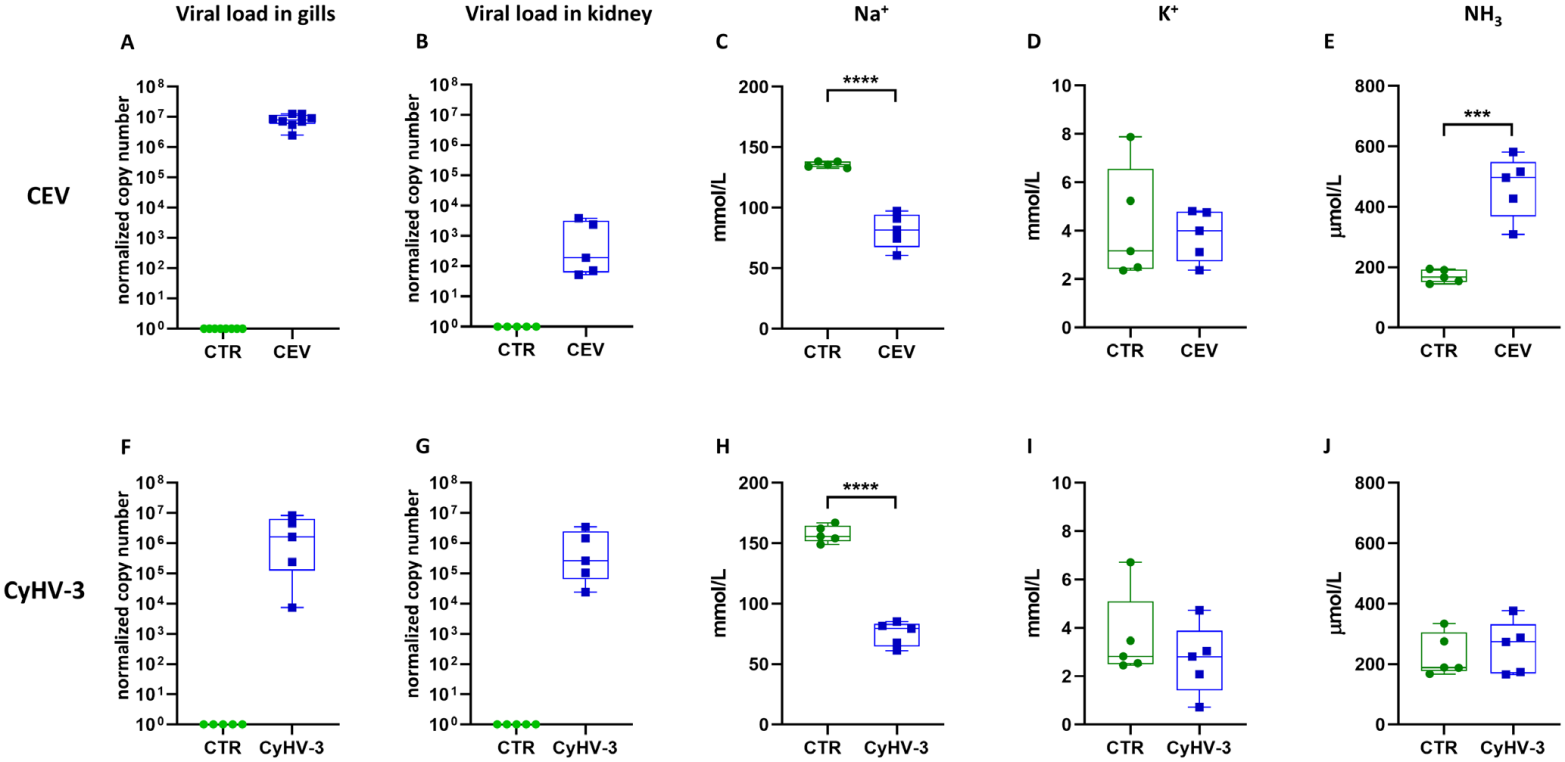
Infection outcome during CEV and CyHV‐3 infection. Viral load presented as normalised copy number of viral DNA per 250 ng of DNA isolated from gills and kidney of fish infected with CEV (A; B) and CyHV‐3 (F; G) compared to uninfected control (CTR). Concentration of ions Na^+^ and K^+^ in blood plasma of CEV infected fish (C; D) and CyHV‐3 infected fish (H; I) compared to uninfected control (CTR). Concentration of NH_3_ in blood plasma of CEV infected fish (E) and CyHV‐3 infected fish (J) compared to uninfected control (CTR). Statistical analysis was performed by Student's *t*‐test or Mann–Whitney test. Asterisks indicate statistically significant differences between control and infected fish (***p ≤ 0.001; ****p ≤ 0.0001). The data are presented as *n* = 8 (CEV) or *n* = 5 (CyHV‐3) individual values on box and whiskers plot.

Infection was confirmed with probe‐based RT‐qPCR in order to quantify viral load. Gills of CEV infected fish had mean 8.22 × 10^6^ viral DNA copies per 250 ng of total DNA isolated. CyHV‐3 infected fish had mean 2.97 × 10^6^ viral DNA copies (Figure 3). Both CEV and CyHV‐3 infections significantly decreased Na^+^ concentration, while CEV also significantly increased ammonia concentration in the blood plasma. K^+^ level in the blood plasma was not affected by the viruses (Figure 3).

### 3.4 NKA Gene Expression in Gills and Kidney Upon CEV and CyHV‐3 Infection

**FIGURE 4:**
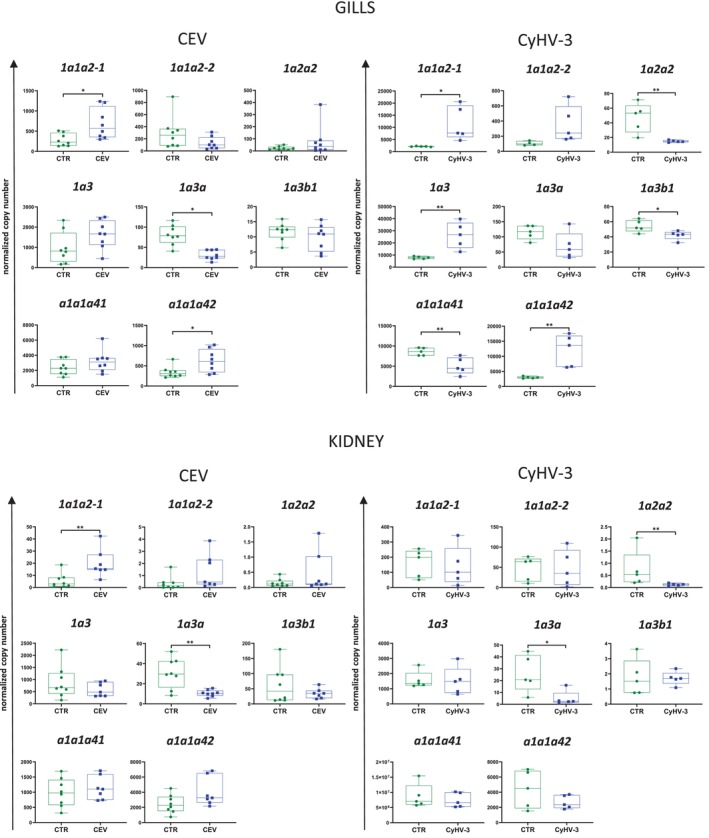
Expression of genes encoding α subunits of Na^+^/K^+^‐ATPases in gills and kidney of control (CTR) and CEV infected fish (CEV) and control (CTR) and CyHV‐3 infected fish (CyHV‐3). Results are presented as the copy number of mRNA of a given gene normalised to two reference genes. Statistical analysis was performed by Student's *t*‐test or Mann–Whitney test. Asterisks indicate statistically significant differences between control and infected fish (*p ≤ 0.05, **p ≤ 0.01). The data are presented as *n* = 8 (CEV) or *n* = 5 (CyHV‐3) individual values on box and whiskers plot.

Expression of genes encoding α subunits of NKAs was differently affected during infection with studied viruses. CEV infection induced upregulation of the expression of *1a1a2–1* and *a1a1a42* in gills and *1a1a2–1* in kidney and downregulation of the expression of *1a3a* in gills and kidney. The expression of other studied NKAs was not affected by CEV in both gills and kidney. CyHV‐3 infection altered expression of most of the studied NKAs in gills and only two in kidney. In gills, CyHV‐3 upregulated the expression of *1a1a2–1*, *1a3* and *a1a1a42* and downregulated the expression of *1a2a2*, *1a3b1* and *a1a1a41*. In kidney, CyHV‐3 downregulated the expression of *1a2a2* and *1a3a* (Figure 4).

### 3.5 Ammonia Transporters Gene Expression in Gills and Kidney Upon CEV and CyHV‐3 Infection

**FIGURE 5:**
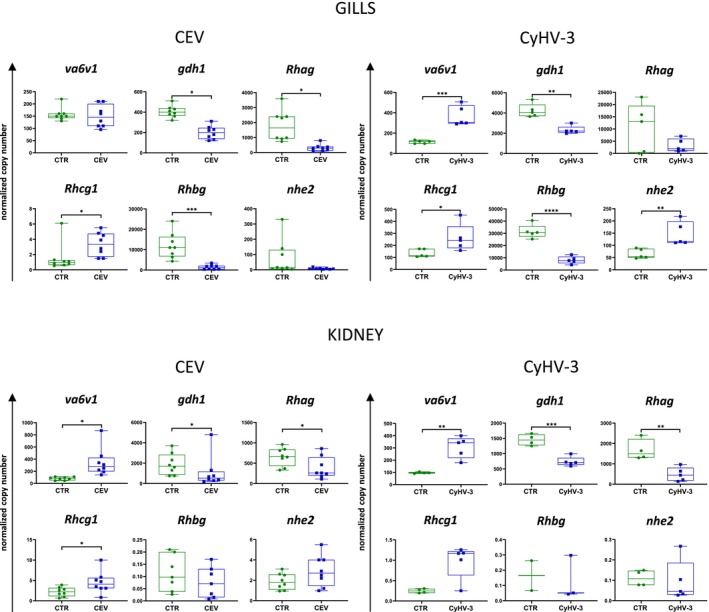
Expression of genes encoding ammonia transporters in gills and kidney of control (CTR) and CEV infected fish (CEV) and control (CTR) and CyHV‐3 infected fish (CyHV‐3). Results are presented as the copy number of mRNA of a given gene normalised to two reference genes. Statistical analysis was performed by Student's *t*‐test or Mann–Whitney test. Asterisks indicate statistically significant differences between control and infected fish (*p ≤ 0.05, **p ≤ 0.01, ***p ≤ 0.001, ****p ≤ 0.0001). The data are presented as *n* = 8 (CEV) or *n* = 5 (CyHV‐3) individual values on box and whiskers plot.

CEV infection induced significant upregulation of the expression of *Rhcg1* and downregulation of *gdh1*, *Rhag* and *Rhbg* in gills. In the kidney, CEV upregulated the expression of *Rhcg1* and *va6v1* and downregulated the expression of *gdh1* and *Rhag*. In the case of CyHV‐3, three ammonia transporters were upregulated in gills (*Rhcg1*, *va6v1* and *nhe2*) and only one in the kidney (*va6v1*). CyHV‐3 also downregulated *gdh1* and *Rhbg* in gills, and similarly to CEV downregulated *gdh1* and *Rhag* in the kidney (Figure 5).

### 3.6 Immunofluorescent Staining of NKA in Gills of CEV and CyHV‐3 Infected Fish

**FIGURE 6:**
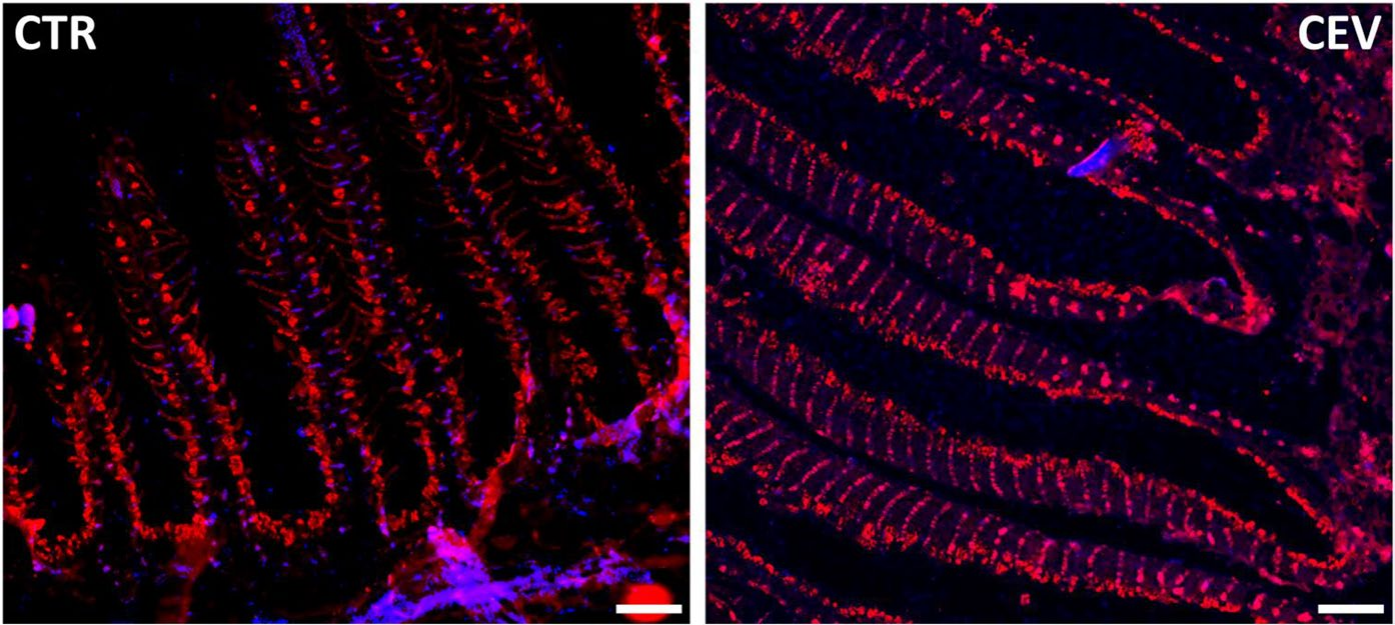
Immunofluorescent staining of Na^+^/K^+^‐ATPases (red) and nucleus (blue) in gills of control (CTR) and CEV infected (CEV) fish. Bar 0.1 mm.

**FIGURE 7:**
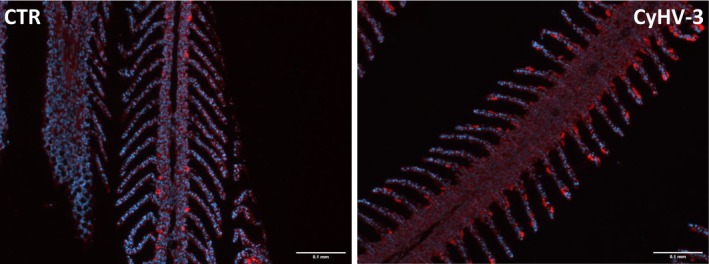
Immunofluorescent staining of Na^+^/K^+^‐ATPases (red) and nucleus (blue) in gills of control (CTR) and CyHV‐3 infected (CyHV‐3) fish. Bar 0.1 mm.

In control fish, NKA‐rich cells were located on the primary lamellae between secondary lamellae. CEV infection resulted in occlusion of intralamellar spaces and transfer of the NKA‐rich cells to the surface of intralammellar cell mass (Figure 6). In fish infected with CyHV‐3, NKA‐rich cells were transferred to the secondary lamellae of the gills (Figure 7).

### 3.7 ATPase Activity

**FIGURE 8:**
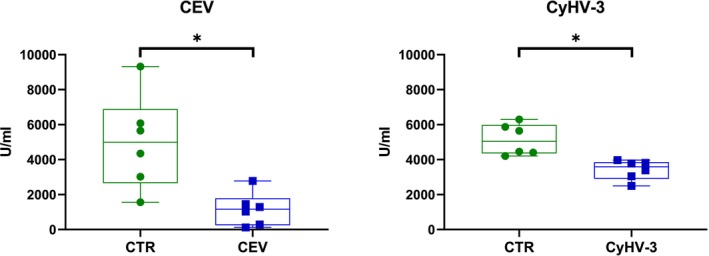
Na^+^/K^+^‐ATPases activity upon CEV infection (A) and CyHV‐3 (B) infection compared to not‐infected control (CTR). Asterisks indicate statistically significant differences between control and infected fish (*p ≤ 0.05). The data are presented as *n* = 8 (CEV) or *n* = 6 (CyHV‐3) individual values on box and whiskers plot.

ATPase activity was analysed using the method by McCormick (McCormick 1993). Mean NKA activity in control fish was 4996.25 U/mL (CEV experiment) or 5146.25 U/mL (for CyHV‐3 experiment). Upon viral infection, NKA activity was significantly reduced to 1160.42 U/mL in the case of CEV and to 3415.75 U/mL in the case of CyHV‐3 (Figure 8).

## 4 Discussion

Osmoregulation and ammonia removal are among key physiological processes that take place in gills and affect fish homeostasis and well‐being. These processes can be disrupted by numerous environmental factors, but also by infections, such as those caused by bacteria *Vibrio* sp. (Deane and Woo 2005) and *Providencia rettgeri* (Baldissera et al. 2019) or parasites such as salmon lice ( *Lepeophtheirus salmonis* ) (Fjelldal et al. 2020; Meyer et al. 2002). Osmoregulation and ammonia removal can also be disrupted during viral infections, especially those leading to severe gill disorders, e.g., fish poxviruses including carp edema virus (Adamek et al. 2021; Zawisza, Chadzinska et al. 2024; Zawisza, Rebl et al. 2024) or salmon gill poxvirus (Gjessing et al. 2017; Nylund et al. 2008). However, the mechanisms underlying the disruption of osmoregulation and ammonia removal during viral infections remain poorly understood.

Previous studies have indicated disruption of osmoregulation, as evidenced by lowered sodium concentrations in the blood during infection with CEV (Adamek et al. 2021) and CyHV‐3 (Negenborn et al. 2015). Therefore, we conducted a further examination of the mechanisms underlying this process, with a particular focus on the NKA. The activity assay demonstrated a decrease in NKA activity in the gills of fish infected with both viruses. Additionally, we examined the expression of genes encoding α subunit of NKA; however, no discernible trends were observed in the expression of all studied genes in the gills and kidneys. In the gills of fish infected with CEV or CyHV‐3, there was an upregulation of *a1a1a42* and *1a1a2–1* expression. In contrast, a significant downregulation of *1a3a* expression was observed in the gills and kidneys of CEV‐infected fish, as well as in the kidneys of CyHV‐3‐infected fish. These findings suggest that NKA α subunit genes are subject to modulation by these infections. However, the direction and magnitude of these changes do not always explain the observed pathological state.

Consistent with our observations, decreased activity of NKA was also revealed during bacterial infection with *Vibrio* sp. in goldlined sea bream ( *Rhabdosargus sarba* ) (Deane and Woo 2005) or with *Providencia rettgeri* in Nile tilapia (Baldissera et al. 2019). Interestingly, *Vibrio* sp. infection in goldlined sea bream did not affect the expression levels of genes encoding α or β subunits of NKA, as determined by semi‐quantitative RT‐PCR coupled with Southern blotting. Nevertheless, changes responsible for the decreased activity were observed at the protein level (Deane and Woo 2005). As the infection progressed, protein concentrations of both subunits of NKA decreased. Specifically, the concentration of subunit α decreased during the late stage of the infection, while the concentration of subunit β decreased from the early stage of infection onwards. The authors hypothesised that the decrease of activity of NKA was a result of either blockade of translation of β subunit or specific degradation of the β subunit in the cells (Deane and Woo 2005), as the role of the β subunit is critical for stability, correct assembly, and proper insertion of the NKA heterodimers into the plasma membrane (Deane and Woo 2005; Geering et al. 1989; Noguchi et al. 1990). In the case of Atlantic salmon (*Salno salar*) infection with salmon gill poxvirus (SGPV), transcriptomic studies have revealed upregulation of the expression of NKA α‐2 subunit and downregulation of the expression of NKA β‐2a subunit in gills (Gjessing et al. 2020). Additionally, there was a decline in the expression of the gene encoding the Na^+^/K^+^/2Cl^−^ co‐transporter (Gjessing et al. 2020). Although the activity of NKA was so far not studied during SGPV infection, histopathology revealed dislocation and hypertrophy of chloride cells (Gjessing et al. 2020). While our present studies concentrated on α or α‐like subunits, further examination is required to elucidate the impact of CEV and CyHV‐3 infection on the β subunits of NKA in common carp. Additionally, examining ATPases at the protein level, as previously mentioned in the context of vibriosis (Deane and Woo 2005), could provide insights into the potential degradation or blockade of specific units of NKA during viral infections.

It is important to note that there are other mechanisms, beyond changes in NKA gene expression and protein level, that can influence blood sodium concentrations. Previous studies have hypothesised that passive loss of ions due to a loosening of the epithelial barrier may be responsible for this process (Adamek et al. 2021). Additionally, the influx of water due to loosed epithelium could also contribute to a decrease in sodium level in the blood. During CyHV‐3 infection, changes in expression of tight junction proteins, which are responsible for maintaining the integrity of the epithelial barrier, such as claudins, were observed in skin and gut (Adamek et al. 2013; Syakuri et al. 2013). CyHV‐3‐induced downregulation of expression of *cldn23* and *cldn30* was observed in skin (Adamek et al. 2013). In gut, downregulation of *cldn3c* was observed, while other claudins were upregulated (Syakuri et al. 2013). In CEV‐infected fish, expression of tight junction proteins (*cdh1* – e‐cadherin and *ocldn a* – occludin a) in gills was not changed (Adamek et al. 2021).

Gills are a target for the stress‐related hormones: cortisol and catecholamines, which have also been shown to affect osmoregulation in fish. In general, cortisol is bound by intracellular glucocorticoid (GR) receptors (Greenwood et al. 2003; Bury and Sturm 2007); however, in fish, it can also be bound by the mineralocorticoid receptor (MR) McCormick et al. (2008). Upon binding to GRs and/or MRs, cortisol seems to be involved not only in the stress response but also in osmoregulation by induction of NKA enzyme activity in gills (as measured by the increased NKA activity and upregulation of *nkaα1a* and *nkaα1b* expression) (McCormick et al. 2008). Increased NKA activity was also observed in the gills of Atlantic salmon during infection with sea lice (Nolan et al. 1999), an ectoparasite that has been reported to induce a high level of plasma cortisol in infected fish (Grimnes and Jakobsen 1996; Tveiten et al. 2010; Wells et al. 2007). There was a positive correlation between plasma cortisol and plasma Na^+^, Cl^−^ levels and osmolality in infected fish, leading to extreme life‐threatening sodium levels in the blood (Fjelldal et al. 2020). Nevertheless, the authors speculated that the elevated plasma cortisol level produced in response to stress induced by sea lice infection may also have a protective response to regulate ion balance by inducing NKA activity (Fjelldal et al. 2020). In the case of CEV infection, a high level of blood plasma cortisol was observed (Zawisza, Rebl et al. 2024); however, NKA activity and the concentration of Na^+^ in blood plasma were decreased. Catecholamines are also produced under stressful conditions and can also affect osmoregulation, but in a different manner. In stressed freshwater fish, catecholamine release was one of the causes of passive ion loss (Randall et al. 1972; Wendelaar Bonga 1997). However, catecholamine levels have not been studied during either CEV or CyHV‐3 infection, so this relationship cannot yet be established.

Gills exhibit a high degree of plasticity, enabling them to respond promptly to changing environmental conditions, stressors and pathogens. The study of gill architecture, with a particular focus on the morphology of ionocytes, has predominantly been conducted in the context of environmental changes, such as variations in temperature, oxygen levels and salinity (Hwang et al. 2011; Sackville and Brauner 2018). The response to infection, however, remains to be explored in greater depth. Our IHC studies showed that during infection with CEV and CyHV‐3, NKA‐rich cells are transferred from the primary lamellae, which is the typical location for chloride cells, to the more superficial space and to the secondary lamellae. The changes in localisation could be related to the gill pathology—e.g., proliferation of the cells or accumulation of the cells debris in the intralamellar spaces leading to their occlusion, which are often visible in the case of CEV (Adamek et al. 2021; Zawisza, Rebl et al. 2024) and aligning with gill pathology visible in current IHC observations. Morphological changes of gill ionocytes during infection may occur to counteract the effect of the pathogen, as previous studies proved they can respond to different environmental conditions in order to maintain ion homeostasis. Experiments on tilapia embryos and larvae indicated that both freshwater‐to‐saltwater and saltwater‐to‐freshwater transitions were mainly associated with functional and morphological changes in pre‐existing ionocytes, demonstrating the ability to adapt to different conditions of unstable water environments (Hiroi et al. 1999). Atlantic salmon can switch ionocyte types during smoltification, as there is a sudden need to excrete ions instead of absorbing them (Hiroi and McCormick 2012). Meanwhile, in rainbow trout, transfer from tap water to artificial soft water (with lower ions concentrations) resulted in proliferation of ionocytes (Greco et al. 1995). In our study, there is no visible decrease in the number of NKA‐rich cells. This contrasts with well‐studied parasitic gill infections; e.g., during amoebic gill disease in Atlantic salmon, there was a decrease in the number of NKA‐rich cells (Chang et al. 2019). Similarly, in Van fish ( *Alburnus tarichi* ) the infection with parasitic myxosporean led to a lower density of NKA positive cells along 500 μm length of gill filament (Oğuz and Oğuz 2020). These results could suggest that gill ionocytes undergo distinct changes in their morphology, function, localisation and number depending on the nature of the stressor, potentially as a compensatory mechanism to maintain fish homeostasis. Understanding these adaptive responses could provide valuable insights into gill pathophysiology and host defence strategies in aquatic species.

CEV infection also led to disruption of ammonia excretion, resulting in accumulation of toxic levels of ammonia (Adamek et al. 2021). In previous studies, it was suggested that the mechanism directly responsible for high ammonia concentration during the CEV infection was the loss of the branchial surface area (Adamek et al. 2021). In the present study, we analysed gene expression of selected ammonia transporters. Both CEV and CyHV‐3 infection resulted in downregulation of the expression of major transporters *gdh1*, *Rhag*, and *Rhbg*, allowing us to partly explain the ammonia accumulation. Signs of compensation could be noticed for certain transporters as upregulation of the expression of *Rhcg1 and va6v1* in CEV‐infected fish, and *Rhcg1, va6v1a* and *nhe2* in CyHV‐3‐infected fish were observed. Indeed, it was previously shown that other Rh transporters may take over the role of Rhbg when the genes are knocked down (Grishin et al. 2020) possibly explaining the pattern in changes of expression of ammonia transporters in our studies. Downregulation of the expression of Rhesus factors was also observed in the gills of Florida pompano ( *Trachinotus carolinus* ) infected with the parasite *Amyloodinium ocellatum*, among the downregulation of the urea transporter. However, the pathological consequences of this parasitic infection were predominantly attributed by the authors to oxidative stress, which was not measured in the present studies (Zhang et al. 2022). In the case of the studied viral infections, the pathophysiology involves high ammonia accumulation; therefore, we postulate that alterations in ammonia transporters play a significant role alongside impaired ion uptake and excretion in the observed clinical signs.

To summarise our study, we observed that two highly important viruses for carp aquaculture (CEV and CyHV‐3) affect NKA activity and expression of ammonia transporters. The results obtained in this study offer valuable insights into the mechanisms underpinning the observed pathophysiology. Disruption to osmoregulation and ammonia excretion has the potential to result in fatal consequences, thus highlighting the critical importance of NKA and ammonia transporters in maintaining homeostatic balance between the fish body and its surrounding aquatic environment.

## Author Contributions

**Maria Zawisza:** investigation, writing – original draft, methodology, visualization, writing – review and editing, data curation. **Muhammad Abdullah:** investigation, writing – review and editing, methodology, visualization, data curation. **Magdalena Marcinkowska:** investigation, writing – review and editing, methodology. **Marek Matras:** investigation, methodology, writing – review and editing. **Krzysztof Rakus:** conceptualization, funding acquisition, writing – original draft, writing – review and editing, visualization, formal analysis, project administration, data curation, supervision, resources. **Mikolaj Adamek:** conceptualization, funding acquisition, methodology, writing – original draft, writing – review and editing, visualization, formal analysis, project administration, data curation, supervision, resources.

## Conflicts of Interest

The authors declare no conflicts of interest.

## Supporting information


Table S1.

## Data Availability

All data are deposited in RODBUK Cracow Open Research Data Repository (https://rodbuk.pl) under DOI: https://doi.org/10.57903/UJ/Y5CAPG.

## References

[jfd14133-bib-0001] Adamek, M. , M. Matras , A. Rebl , et al. 2022. “Don't Let It Get Under Your Skin!—Vaccination Protects the Skin Barrier of Common Carp From Disruption Caused by Cyprinid Herpesvirus 3.” Frontiers in Immunology 13: 787021. 10.3389/fimmu.2022.787021. 35173716 PMC8842664

[jfd14133-bib-0002] Adamek, M. , A. Oschilewski , P. Wohlsein , et al. 2017. “Experimental Infections of Different Carp Strains With the Carp Edema Virus (CEV) Give Insights Into the Infection Biology of the Virus and Indicate Possible Solutions to Problems Caused by Koi Sleepy Disease (KSD) in Carp Aquaculture.” Veterinary Research 48, no. 1: 12. 10.1186/s13567-017-0416-7. 28222784 PMC5320791

[jfd14133-bib-0003] Adamek, M. , H. Syakuri , S. Harris , et al. 2013. “Cyprinid Herpesvirus 3 Infection Disrupts the Skin Barrier of Common Carp (*Cyprinus carpio* L.).” Veterinary Microbiology 162, no. 2–4: 456–470. 10.1016/j.vetmic.2012.10.033. 23182910

[jfd14133-bib-0004] Adamek, M. , F. Teitge , I. Baumann , et al. 2021. “Koi Sleepy Disease as a Pathophysiological and Immunological Consequence of a Branchial Infection of Common Carp With Carp Edema Virus.” Virulence 12, no. 1: 1855–1883. 10.1080/21505594.2021.1948286. 34269137 PMC8288041

[jfd14133-bib-0005] Baldissera, M. D. , C. F. Souza , S. N. Descovi , et al. 2019. “Impairment of Branchial Energy Transfer Pathways in Disease Pathogenesis of *Providencia rettgeri* Infection in Juvenile Nile Tilapia ( *Oreochromis niloticus* ): Remarkable Involvement of Creatine Kinase Activity.” Aquaculture 502: 365–370. 10.1016/j.aquaculture.2018.12.074.

[jfd14133-bib-0006] Blanco, G. , and R. W. Mercer . 1998. “Isozymes of the Na‐K‐ATPase: Heterogeneity in Structure, Diversity in Function.” American Journal of Physiology. Renal Physiology 275, no. 5: F633–F650. 10.1152/ajprenal.1998.275.5.F633. 9815123

[jfd14133-bib-0007] Boutier, M. , Y. Gao , O. Donohoe , and A. Vanderplasschen . 2019. “Current Knowledge and Future Prospects of Vaccines Against Cyprinid Herpesvirus 3 (CyHV‐3).” Fish & Shellfish Immunology 93: 531–541. 10.1016/j.fsi.2019.07.079. 31369858

[jfd14133-bib-0008] Boutier, M. , M. Ronsmans , K. Rakus , et al. 2015. “Cyprinid Herpesvirus 3: An Archetype of Fish Alloherpesviruses.” Advances in Virus Research 93: 161–256. 10.1016/bs.aivir.2015.03.001. 26111587

[jfd14133-bib-0009] Bury, N. R. , and A. Sturm . 2007. “Evolution of the Corticosteroid Receptor Signalling Pathway in Fish.” General and Comparative Endocrinology 153, no. 1–3: 47–56. 17470371 10.1016/j.ygcen.2007.03.009

[jfd14133-bib-0010] Chang, Y. , H. Hamlin‐Wright , S. Monaghan , et al. 2019. “Changes in Distribution, Morphology and Ultrastructure of Chloride Cell in Atlantic Salmon During an AGD Infection.” Journal of Fish Diseases 42, no. 10: 1433–1446. 10.1111/jfd.13073. 31429104

[jfd14133-bib-0011] Deane, E. E. , and N. Y. S. Woo . 2005. “Evidence for Disruption of Na‐K‐ATPase and hsp70 During Vibriosis of Sea Bream, Sparus (=Rhabdosargus) Sarba Forsskål.” Journal of Fish Diseases 28, no. 4: 239–251. 10.1111/j.1365-2761.2005.00624.x. 15813866

[jfd14133-bib-0012] Dereeper, A. , V. Guignon , G. Blanc , et al. 2008. “Phylogeny.fr: Robust Phylogenetic Analysis for the Non‐Specialist.” Nucleic Acids Research 36: W465‐9. 18424797 10.1093/nar/gkn180 PMC2447785

[jfd14133-bib-0013] Einarson, S. 1993. “Effects of Temperature, Seawater Osmolality and Season on Oxygen Consumption and Osmoregulation of the Amphipod *Gammarus oceanicus* .” Marine Biology 117, no. 4: 599–606. 10.1007/BF00349771.

[jfd14133-bib-0014] Evans, D. H. , P. M. Piermarini , and K. P. Choe . 2005. “The Multifunctional Fish Gill: Dominant Site of Gas Exchange, Osmoregulation, Acid‐Base Regulation, and Excretion of Nitrogenous Waste.” Physiological Reviews 85, no. 1: 97–177. 10.1152/physrev.00050.2003. 15618479

[jfd14133-bib-0015] Fjelldal, P. G. , T. J. Hansen , and Ø. Karlsen . 2020. “Effects of Laboratory Salmon Louse Infection on Osmoregulation, Growth and Survival in Atlantic Salmon.” Conservation Physiology 8, no. 1: coaa023. 10.1093/conphys/coaa023. 32257215 PMC7098368

[jfd14133-bib-0016] Fu, C. , J. M. Wilson , P. J. Rombough , and C. J. Brauner . 2010. “Ions First: Na+ Uptake Shifts From the Skin to the Gills Before O_2_ Uptake in Developing Rainbow Trout, *Oncorhynchus mykiss* .” Proceedings of the Royal Society B: Biological Sciences 277, no. 1687: 1553–1560. 10.1098/rspb.2009.1545. PMC2871827 20071386

[jfd14133-bib-0017] Geering, K. , I. Theulaz , F. Verrey , M. T. Hauptle , and B. C. Rossier . 1989. “A Role for the Beta‐Subunit in the Expression of Functional Na+‐K+‐ATPase in Xenopus Oocytes.” American Journal of Physiology‐Cell Physiology 257, no. 5: C851–C858. 10.1152/ajpcell.1989.257.5.C851. 2556932

[jfd14133-bib-0018] Gilad, O. , S. Yun , F. Zagmutt‐Vergara , C. Leutenegger , H. Bercovier , and R. Hedrick . 2004. “Concentrations of a Koi Herpesvirus (KHV) in Tissues of Experimentally‐Infected *Cyprinus carpio* koi as Assessed by Real‐Time TaqMan PCR.” Diseases of Aquatic Organisms 60: 179–187. 10.3354/dao060179. 15521316

[jfd14133-bib-0019] Gjessing, M. C. , A. Krasnov , G. Timmerhaus , et al. 2020. “The Atlantic Salmon Gill Transcriptome Response in a Natural Outbreak of Salmon Gill Poxvirus Infection Reveals New Biomarkers of Gill Pathology and Suppression of Mucosal Defense.” Frontiers in Immunology 11: 2154. 10.3389/fimmu.2020.02154. 33013908 PMC7509425

[jfd14133-bib-0020] Gjessing, M. C. , E. Thoen , T. Tengs , S. A. Skotheim , and O. B. Dale . 2017. “Salmon Gill Poxvirus, a Recently Characterized Infectious Agent of Multifactorial Gill Disease in Freshwater‐ and Seawater‐Reared Atlantic Salmon.” Journal of Fish Diseases 40, no. 10: 1253–1265. 10.1111/jfd.12608. 28105681

[jfd14133-bib-0021] Greco, A. M. , K. M. Gilmour , J. C. Fenwick , and S. F. Perry . 1995. “The Effects of Softwater Acclimation on Respiratory Gas Transfer in the Rainbow Trout *Oncorhynchus mykiss* .” Journal of Experimental Biology 198, no. 12: 2557–2567. 10.1242/jeb.198.12.2557. 9320486

[jfd14133-bib-0022] Greenwood, A. K. , P. C. Butler , R. B. White , U. DeMarco , D. Pearce , and R. D. Fernald . 2003. “Multiple Corticosteroid Receptors in a Teleost Fish: Distinct Sequences, Expression Patterns, and Transcriptional Activities.” Endocrinology 144, no. 10: 4226–4236. 10.1210/en.2003-0566. 12959971

[jfd14133-bib-0023] Grimnes, A. , and P. J. Jakobsen . 1996. “The Physiological Effects of Salmon Lice Infection on Post‐Smolt of Atlantic Salmon.” Journal of Fish Biology 48, no. 6: 1179–1194. 10.1111/j.1095-8649.1996.tb01813.x.

[jfd14133-bib-0024] Grishin, D. V. , E. Y. Kasap , A. A. Izotov , and A. V. Lisitsa . 2020. “Multifaceted Ammonia Transporters.” All Life 13, no. 1: 486–497. 10.1080/26895293.2020.1812443.

[jfd14133-bib-0025] Handeland, S. O. , Å. Berge , B. T. Björnsson , and S. O. Stefansson . 1998. “Effects of Temperature and Salinity on Osmoregulation and Growth of Atlantic Salmon ( *Salmo salar* L.) Smolts in Seawater.” Aquaculture 168, no. 1–4: 289–302. 10.1016/S0044-8486(98)00356-1.

[jfd14133-bib-0026] Hiroi, J. , T. Kaneko , and M. Tanaka . 1999. “In Vivo Sequential Changes in Chloride Cell Morphology in the Yolk‐Sac Membrane of Mozambique Tilapia ( *Oreochromis mossambicus* ) Embryos and Larvae During Seawater Adaptation.” Journal of Experimental Biology 202, no. 24: 3485–3495. 10.1242/jeb.202.24.3485. 10574727

[jfd14133-bib-0027] Hiroi, J. , and S. D. McCormick . 2012. “New Insights Into Gill Ionocyte and Ion Transporter Function in Euryhaline and Diadromous Fish.” Respiratory Physiology & Neurobiology 184, no. 3: 257–268. 10.1016/j.resp.2012.07.019. 22850177

[jfd14133-bib-0028] Hwang, P.‐P. , T.‐H. Lee , and L.‐Y. Lin . 2011. “Ion Regulation in Fish Gills: Recent Progress in the Cellular and Molecular Mechanisms.” American Journal of Physiology. Regulatory, Integrative and Comparative Physiology 301, no. 1: R28–R47. 10.1152/ajpregu.00047.2011. 21451143

[jfd14133-bib-0029] Jung‐Schroers, V. , M. Adamek , F. Teitge , et al. 2015. “Another Potential Carp Killer?: Carp Edema Virus Disease in Germany.” BMC Veterinary Research 11, no. 1: 1–4. 10.1186/s12917-015-0424-7. 25976542 PMC4431602

[jfd14133-bib-0030] Lemaire, P. , E. Bernard , J. A. Martinez‐Paz , and L. Chim . 2002. “Combined Effect of Temperature and Salinity on Osmoregulation of Juvenile and Subadult *Penaeus stylirostris* .” Aquaculture 209, no. 1–4: 307–317. 10.1016/S0044-8486(01)00756-6.

[jfd14133-bib-0031] Machat, R. , L. Pojezdal , V. Piackova , and M. Faldyna . 2021. “Carp Edema Virus and Immune Response in Carp ( *Cyprinus carpio* ): Current Knowledge.” Journal of Fish Diseases 44: 371–378. 10.1111/jfd.13335. 33460151

[jfd14133-bib-0032] Marshall, W. S. 2002. “Na+, Cl−, Ca2+ and Zn2+ Transport by Fish Gills: Retrospective Review and Prospective Synthesis.” Journal of Experimental Zoology 293, no. 3: 264–283. 10.1002/jez.10127. 12115901

[jfd14133-bib-0033] Matras, M. , E. Borzym , D. Stone , et al. 2017. “Carp Edema Virus in Polish Aquaculture—Evidence of Significant Sequence Divergence and a New Lineage in Common Carp *Cyprinus carpio* (L.).” Journal of Fish Diseases 40, no. 3: 319–325. 10.1111/jfd.12518. 27453481

[jfd14133-bib-0034] McCormick, S. D. 1993. “Methods for Nonlethal Gill Biopsy and Measurement of Na+, K+‐ATPase Activity.” Canadian Journal of Fisheries and Aquatic Sciences 50, no. 3: 656–658.

[jfd14133-bib-0035] McCormick, S. D. , A. Regish , M. F. O'Dea , and J. M. Shrimpton . 2008. “Are We Missing a Mineralocorticoid in Teleost Fish? Effects of Cortisol, Deoxycorticosterone and Aldosterone on Osmoregulation, Gill Na+, K+‐ATPase Activity and Isoform mRNA Levels in Atlantic Salmon.” General and Comparative Endocrinology 157, no. 1: 35–40. 18462736 10.1016/j.ygcen.2008.03.024

[jfd14133-bib-0036] Meyer, C. , M. Ganter , W. Körting , and D. Steinhagen . 2002. “Effects of a Parasite‐Induced Nephritis on Osmoregulation in the Common Carp *Cyprinus carpio* .” Diseases of Aquatic Organisms 50: 127–135. 10.3354/dao050127. 12180703

[jfd14133-bib-0037] Miwa, S. , I. Kiryu , K. Yuasa , T. Ito , and T. Kaneko . 2015. “Pathogenesis of Acute and Chronic Diseases Caused by Cyprinid Herpesvirus‐3.” Journal of Fish Diseases 38, no. 8: 695–712. 10.1111/jfd.12282. 25073413

[jfd14133-bib-0038] Miyazaki, T. , T. Isshiki , and H. Katsuyuki . 2005. “Histopathological and Electron Microscopy Studies on Sleepy Disease of Koi *Cyprinus carpio* koi in Japan.” Diseases of Aquatic Organisms 65, no. 3: 197–207. 10.3354/dao065197. 16119888

[jfd14133-bib-0039] Miyazaki, T. , Y. Kuzuya , S. Yasumoto , M. Yasuda , and T. Kobayashi . 2008. “Histopathological and Ultrastructural Features of Koi Herpesvirus (KHV)‐Infected Carp *Cyprinus carpio*, and the Morphology and Morphogenesis of KHV.” Diseases of Aquatic Organisms 80: 1–11. 10.3354/dao01929. 18714678

[jfd14133-bib-0040] Morth, J. P. , B. P. Pedersen , M. J. Buch‐Pedersen , et al. 2011. “A Structural Overview of the Plasma Membrane Na+K+‐ATPase and H+‐ATPase Ion Pumps.” Nature Reviews Molecular Cell Biology 12, no. 1: 60–70. 10.1038/nrm3031. 21179061

[jfd14133-bib-0041] Negenborn, J. , M. C. van der Marel , M. Ganter , and D. Steinhagen . 2015. “Cyprinid Herpesvirus‐3 (CyHV‐3) Disturbs Osmotic Balance in Carp ( *Cyprinus carpio* L.)—A Potential Cause of Mortality.” Veterinary Microbiology 177, no. 3–4: 280–288. 10.1016/j.vetmic.2015.03.018. 25888311

[jfd14133-bib-0042] Noguchi, S. , K. Higashi , and M. Kawamura . 1990. “A Possible Role of the Beta‐Subunit of (Na,K)‐ATPase in Facilitating Correct Assembly of the Alpha‐Subunit Into the Membrane.” Journal of Biological Chemistry 265, no. 26: 15991–15995. 10.1016/S0021-9258(18)55495-8. 2168428

[jfd14133-bib-0043] Nolan, D. T. , P. Reilly , and S. W. Bonga . 1999. “Infection With Low Numbers of the Sea Louse *Lepeophtheirus salmonis* Induces Stress‐Related Effects in Postsmolt Atlantic Salmon (*Salmo salar*).” Canadian Journal of Fisheries and Aquatic Sciences 56, no. 6: 947–959. 10.1139/f99-021.

[jfd14133-bib-0044] Nylund, A. , K. Watanabe , S. Nylund , et al. 2008. “Morphogenesis of Salmonid Gill Poxvirus Associated With Proliferative Gill Disease in Farmed Atlantic Salmon ( *Salmo salar* ) in Norway.” Archives of Virology 153, no. 7: 1299–1309. 10.1007/s00705-008-0117-7. 18521535

[jfd14133-bib-0045] Oğuz, A. R. , and E. K. Oğuz . 2020. “Histopathology and Immunohistochemistry of Gills of Van Fish ( *Alburnus tarichi* Güldenstädt, 1814) Infected With Myxosporean Parasites.” Journal of Histotechnology 43, no. 2: 76–82. 10.1080/01478885.2019.1686848. 31783723

[jfd14133-bib-0046] Pikarsky, E. , A. Ronen , J. Abramowitz , et al. 2004. “Pathogenesis of Acute Viral Disease Induced in Fish by Carp Interstitial Nephritis and Gill Necrosis Virus.” Journal of Virology 78, no. 17: 9544–9551. 10.1128/JVI.78.17.9544-9551.2004. 15308746 PMC506927

[jfd14133-bib-0047] Pikula, J. , L. Pojezdal , I. Papezikova , et al. 2021. “Carp Edema Virus Infection Is Associated With Severe Metabolic Disturbance in Fish.” Frontiers in Veterinary Science 8: 679970. 10.3389/fvets.2021.679970. 34095283 PMC8169968

[jfd14133-bib-0048] Rakus, K. , P. Ouyang , M. Boutier , et al. 2013. “Cyprinid Herpesvirus 3: An Interesting Virus for Applied and Fundamental Research.” Veterinary Research 44, no. 1: 85. 10.1186/1297-9716-44-85. 24073814 PMC3850573

[jfd14133-bib-0049] Randall, D. J. , D. Baumgarten , and M. Malyusz . 1972. “The Relationship Between Gas and Ion Transfer Across the Gills of Fishes.” Comparative Biochemistry and Physiology Part A: Physiology 41, no. 3: 629–637. 10.1016/0300-9629(72)90017-5. 4401733

[jfd14133-bib-0050] Randall, D. J. , and T. K. N. Tsui . 2002. “Ammonia Toxicity in Fish.” Marine Pollution Bulletin 45, no. 1–12: 17–23. 10.1016/S0025-326X(02)00227-8. 12398363

[jfd14133-bib-0051] Rességuier, J. , A. S. Dalum , L. Du Pasquier , et al. 2020. “Lymphoid Tissue in Teleost Gills: Variations on a Theme.” Biology 9, no. 6: 1–14. 10.3390/biology9060127. PMC7344468 32549335

[jfd14133-bib-0052] Rombough, P. 2007. “The Functional Ontogeny of the Teleost Gill: Which Comes First, Gas or Ion Exchange?” Comparative Biochemistry and Physiology Part A: Molecular & Integrative Physiology 148, no. 4: 732–742. 10.1016/j.cbpa.2007.03.007. 17451987

[jfd14133-bib-0053] Sackville, M. A. , and C. J. Brauner . 2018. “Case Study: Gill Plasticity in Larval Fishes.” In Development and Environment, 377–400. Springer International Publishing. 10.1007/978-3-319-75935-7_15.

[jfd14133-bib-0054] Syakuri, H. , M. Adamek , G. Brogden , et al. 2013. “Intestinal Barrier of Carp (*Cyprinus carpio* L.) During a Cyprinid Herpesvirus 3‐Infection: Molecular Identification and Regulation of the mRNA Expression of Claudin Encoding Genes.” Fish & Shellfish Immunology 34, no. 1: 305–314. 10.1016/j.fsi.2012.11.010. 23194746

[jfd14133-bib-0055] Tveiten, H. , P. A. Bjørn , H. K. Johnsen , B. Finstad , and R. S. McKinley . 2010. “Effects of the Sea Louse *Lepeophtheirus salmonis* on Temporal Changes in Cortisol, Sex Steroids, Growth and Reproductive Investment in Arctic Charr *Salvelinus alpinus* .” Journal of Fish Biology 76, no. 10: 2318–2341. 10.1111/j.1095-8649.2010.02636.x. 20557595

[jfd14133-bib-0056] Vargas‐Chacoff, L. , F. J. Arjona , I. Ruiz‐Jarabo , A. García‐Lopez , G. Flik , and J. M. Mancera . 2020. “Water Temperature Affects Osmoregulatory Responses in Gilthead Sea Bream ( *Sparus aurata* L.).” Journal of Thermal Biology 88: 102526. 10.1016/j.jtherbio.2020.102526. 32126001

[jfd14133-bib-0057] Vargas‐Chacoff, L. , A. M. Regish , A. Weinstock , and S. D. McCormick . 2018. “Effects of Elevated Temperature on Osmoregulation and Stress Responses in Atlantic Salmon *Salmo salar* Smolts in Fresh Water and Seawater.” Journal of Fish Biology 93, no. 3: 550–559. 10.1111/jfb.13683. 29956316

[jfd14133-bib-0058] Waltzek, T. B. , G. O. Kelley , D. M. Stone , et al. 2005. “Koi Herpesvirus Represents a Third Cyprinid Herpesvirus (CyHV‐3) in the Family Herpesviridae.” Journal of General Virology 86, no. 6: 1659–1667. 10.1099/vir.0.80982-0. 15914843

[jfd14133-bib-0059] Wells, A. , C. E. Grierson , L. Marshall , et al. 2007. “Physiological Consequences of “Premature Freshwater Return” for Wild Sea‐Run Brown Trout (*Salmo trutta*) Postsmolts Infested With Sea Lice (*Lepeophtheirus salmonis*).” Canadian Journal of Fisheries and Aquatic Sciences 64, no. 10: 1360–1369. 10.1139/f07-107.

[jfd14133-bib-0060] Wendelaar Bonga, S. E. 1997. “The Stress Response in Fish.” Physiological Reviews 77, no. 3: 591–625. 10.1152/physrev.1997.77.3.591. 9234959

[jfd14133-bib-0061] Wright, P. A. , and C. M. Wood . 2009. “A New Paradigm for Ammonia Excretion in Aquatic Animals: Role of Rhesus(Rh) Glycoproteins.” Journal of Experimental Biology 212, no. 15: 2303–2312. 10.1242/jeb.023085. 19617422

[jfd14133-bib-0062] Wright, P. A. , and C. M. Wood . 2012. “Seven Things Fish Know About Ammonia and We Don't.” Respiratory Physiology & Neurobiology 184, no. 3: 231–240. 10.1016/j.resp.2012.07.003. 22910326

[jfd14133-bib-0063] Zawisza, M. , M. Chadzinska , D. Steinhagen , K. Rakus , and M. Adamek . 2024. “Gill Disorders in Fish: Lessons From Poxvirus Infections.” Reviews in Aquaculture 16, no. 1: 234–253. 10.1111/raq.12835.

[jfd14133-bib-0064] Zawisza, M. , A. Rebl , F. Teitge , et al. 2024. “Stressing Out—Carp Edema Virus Induces Stress and Modulates Immune Response in Common Carp.” Frontiers in Immunology 15: 1350197. 10.3389/fimmu.2024.1350197. 38576605 PMC10991768

[jfd14133-bib-0065] Zhang, D. , H. Mohammed , Z. Ye , et al. 2022. “Transcriptomic Profiles of Florida Pompano (*Trachinotus carolinus*) Gill Following Infection by the Ectoparasite *Amyloodinium ocellatum* .” Fish & Shellfish Immunology 125: 171–179. 10.1016/j.fsi.2022.05.017. 35569776

