# Supplementary material for: Modulation of Sodium and Ammonia Transporters in the Context of Viral Gill Diseases in Common Carp (Cyprinus carpio)
Source: J Fish Dis. 2025 Apr 23;48(10):e14133. doi: 10.1111/jfd.14133 (PMC12421795; doi:10.1111/jfd.14133)
Supplement: Supplementary file 1 — Table S1. [file JFD-48-e14133-s001.docx]

Supplementary material

Supplementary table 1. Primers used for RT-qPCR

| **Gene** | **Forward primer sequence** | **Reverse primer sequence** | **Accession number** |
| --- | --- | --- | --- |
| *40S* | CCGTGGGTGACATCGTTACA | TCAGGACATTGAACCTCACTGTCT | AB012087 |
| *ef1a* | ACAACCCCAAGGCTCTCAA | CCGCCAACTTTCTTCTCAAC | AF485331 |
| *CEV p4a* | AGTTTTGTAKATTGTAGCATTTCC | GATTCCTCAAGGAGTTDCAGTAAA | OP494128.1 |
| *CyHV-3 ORF89* | GACGCCGGAGACCTTGTG | CGGGTTCTTATTTTTGTCCTTGTT | AF411803 |
| *Na^+/^K^+^-ATPase 1a1a2-1* | CCTTCCTCATGTTCGTCCTC | TTGAGCATTTCTGGGTTGTC | XM_042717075.1 |
| *Na^+/^K^+^-ATPase 1a1a2-2* | AGCAGTTGTTTTCCTGATTGG | CAGAGCAAATGGTGGTGGTT | XM_042746161.1 |
| *Na^+/^K^+^-ATPase 1a2a2* | TTCCAAGGGGGTTTGAGTTT | CGGCATTTTCCTACAGCATC | XM_042778583.1 |
| *Na^+/^K^+^-ATPase 1a3* | GGAAGGTGGAAAAACACCAA | CCCAGGAAGACAGCTACACC | XM_042717076.1 |
| *Na^+/^K^+^-ATPase 1a3a* | ATGGACGAGGAACTGMGGA | GCATGAACAAGTGGCAGAAA | XM_042746047.1 |
| *Na^+/^K^+^-ATPase 1a3b1* | TGGATAACTCCTCCCTCACC | ATCACCAGTGCAGACCACAA | XM_042772066.1 |
| *Na^+/^K^+^-ATPase a1a1a41* | TTGTCACTGGAGTAGAAGAAGGA | GGTCAATGCAGAGAATGGTG | XM_042717076.1 |
| *Na^+/^K^+^-ATPase a1a1a42* | GAACCCCAGACTCGTACTCCT | GGTGTTGATGACAATCCCTCT | XM_042746459.1 |
| *va6v1* | GCAGCAGAACATCTCCACAA | GAGCATCCATAACACCACAAGA | XM_042714329.1 |
| *gdh1* | TTCCCCATCAAGAGAGACAA | TCACCTCATCCACTGACACC | XM_042736835.1 |
| *Rhag* | GGTGGAGAAGAAGGGGAAAC | GCGAGTATGGGAGTGAGGAA | KC820797.1 |
| *Rhcg1* | CAGAAAGGAGAAGAACATAACGAG | AAACCAAAGCCCACAAAGA | KF051940.1 |
| *Rhbg* | GAATGGCTCTGATTGGTGGT | GAGTTTTTCCGTTTCGCTTG | KF051941.1 |
| *nhe2* | GCTGTGGTTTGCTGTTGTTG | GAGATGATGGAGGCGAAGAG | XM_042730904.1 |
